# Supplementary material for: IL17 factors are early regulators in the gut epithelium during inflammatory response to Vibrio in the sea urchin larva
Source: eLife. 2017 Apr 27;6:e23481. doi: 10.7554/eLife.23481 (PMC5457136; doi:10.7554/eLife.23481)
Supplement: Supplementary file 1. — DOI: http://dx.doi.org/10.7554/eLife.23481.021 [file elife-23481-supp1.docx]

**Supplementary File 1: Sequences of oligonucleotides used for qPCR, RACE, WMISH, and reporter BAC constructs**

| ***Oligonucleotides used for qPCR*** | | | | | | | | |
| --- | --- | --- | --- | --- | --- | --- | --- | --- |
| **Target** | | | | | **Forward sequence** | | | **Reverse sequence** |
| SpIL17-1 | | | | | CATCAAGCTGCCCATACGAT | | | GCTGATCGACATCGGGATAC |
| SpIL17-4 | | | | | CTCTGTCCCAGGAAGCAATA | | | GGTGGCCAGTGGGTCTTC |
| SpIL17-4a | | | | | CACAACTTAGGAGGAAAGCTGAT | | | GCATCAAGCACAGTGCACAA |
| SpIL17-4a´ | | | | | CGGGACACCTAGGCTCTTAAT | | |  |
| SpIL17-9 | | | | | CAATCAGGAGCCTCTCRAGT | | | AGGGTTAATACAATCACGGCAC |
| SpIL17R1 exons 3-4 | | | | | TTCTTTGGCATCATCTTGGA | | | GCTGTTGTAGCCCAGTCCAT |
| SpIL17R1 exons 13-14 | | | | | ATCGTAGAAGGCTGGAAGCA | | | TGGACATGCTCATCACACCT |
| SpIL17R1 exons 14-15 | | | | | AACTGCTCCTGTGTTCCTGGCTAC | | | CCTGGTCTTTAGGATGAACCTGAA |
| SpIL17R1 exons 15-16 | | | | | CAGCATTGCTAGTGGGCTTCAGGTT | | | GAGGAGGTGTAGGTGGTGAT |
| SpIL17R1 exons 15-17 | | | | | CCACCTACACCTCCTCCAGA | | | ATATGATGAGAATCCTCTTCTCGGGT |
| SpIL17R2 | | | | | CATCCTTATTGCTGTCGGTCT | | | TTCTTCGGGATAGGCAAAGA |
| 185/333 | | | | | TGGAGGTGAAAGTGACACTGA | | | CATTCTCCTTTCCTCGTCGTT |
| α2-Macroglobulin (SPU_011257) | | | | | TCTGAATGAGGATGTACAACAGG | | | ACACTTTGCCCATCAGATCC |
| C3 (SPU_005182) | | | | | ATGGATGGCAGCGTTATTG | | | ATCTGCCCATTGGGTTCATA |
| Caspase 3/7 ( SPU_002280) | | | | | CCAGTAGGGCAGCCATCTTG | | | TCGAATCGGTTACCATGGCG |
| Cebpα (SPU_001657) | | | | | TATAAGCAGAAGCGGGAACG | | | TTGGAGCTCCTTGTTCTTCG |
| Cebpγ (SPU_011002) | | | | | GAACTGACACGTACCGTTGC | | | GGAGGCCATGGTGGTATCT |
| Ets4 (SPU_008528) | | | | | CTCCAGCCCAACTCCTACAG | | | GATGGAGCGAGAGAGCTTGT |
| MacpfA (SPU_022091) | | | | | AGCGGCTTGTTTGCTAGTGT | | | GTAACGAGCGTTTCCCATGT |
| Mif7 (SPU_001152) | | | | | GCTGAGGCTTGGTTAGATCG | | | GTCGGGTGAGATACCAAGGA |
| NFκB (SPU_008177) | | | | | ACTTTAGCGGCAACCAAATG | | | CACCGGTCGATGTTGTGTAT |
| NFKBIZ (SPU_024401) | | | | | AGATCGTGGTTTGGAAGACG | | | TTTGGTGCCATGATGGTAGA |
| Socs2/3 (SPU_002792) | | | | | AGGCGAGGAAATGACCACAG | | | GAGAGAATCGAGGCGGTGAG |
| Socs6 (SPU_011298) | | | | | GTCCACATTCAAGCGGAAAT | | | ATCACAAACGTTCCCGTGTT |
| SOUL1 (SPU_003077) | | | | | GGACAAAACGAAGGCAATCA | | | CCTGGGACCATTTTCTGTTG |
| SPU_005664 | | | | | CATAGCCGGGACAGTGTACG | | | GATGGTACCCCGGATTCTCC |
| SPU_022144 | | | | | TCAATGCCACCAAGCCTAA | | | TCCATGGTTCCTTGGGATAG |
| SRCR143 (SPU_018430) | | | | | AGTTCGTTGTCTTGTGCCTGA | | | TCCCCATTCTCCGTTGTAGC |
| Stat (SPU_015108) | | | | | CGGTGCTAGCTAGTGAGACA | | | CGATCTGCTCTGCGAATTCT |
| Tecp2 (SPU_019422) | | | | | GGAGAAAACGTTCCCATCAA | | | CGCAGACATCCTGAGAATCA |
| TNFAIP3 (SPU_000649) | | | | | ACGCACATTCCTGGTCAAG | | | GGCGTTCAGGACTGGAGATA |
| TNFSF1 (SPU_009528) | | | | | CACATGTTGTTCCTCTCATCTCC | | | GGTCCGTCCACACAAACC |
|  | | | | |  | | |  |
| ***Oligonucleotides used for RACE*** | | | | | | | | |
| **Target** | **End** | | | **Primer** | | | **Sequence** | |
| SpIL17-1 | 5´ | | | First amplification | | | CCATGCTGATCGACATCGGGATAC | |
|  |  |  |  | Nested | | | GAGTTGACTGGWTCAGAGGCACTTG | |
|  | 3´ | | | First amplification | | | GAGACATCAAGCTGCCCATACGAT | |
|  |  |  |  | Nested | | | CAAGTGCCTCTGAWCCAGTCAACTC | |
| SpIL17-4 | 5´ | | | First amplification | | | CATGCTAGGGCACGTGCTAA | |
|  |  |  |  | Nested | | | TGCAGCTCTCGGTCGTTCTTC | |
| SpIL17R1 | 5´ | | | First amplification | | | AGCCAGGAACACAGGAGCAGTTG | |
|  |  |  |  | Nested | | | CAGGAACACAGGAGCAGTTGTATCG | |
|  | 3´ | | | First amplification | | | GGTGAATCGTCCCTGGACATCGT | |
|  |  |  |  | Nested | | | GAAGGCTGGAAGCACCATGACTG | |
| SpIL17R2 | 5´ | | | First amplification | | | GTTGAGAGCTCCTTCTTGGCACTGA | |
|  |  |  |  | Nested | | | AACCGAGAGGCTCGCAGGTGTTT | |
|  | 3´ | | | First amplification | | | ACGTCAACACTTGTGGACCTCTTG | |
|  |  |  |  | Nested | | | TGGACCTCTTGGATCCTGCATTC | |
| ***Oligonucleotides used for BAC recombination*** | | | | | | | | |
| **Target** | | **Arm** | **Forward sequence** | | | **Reverse sequence** | | |
| SpIL17-1d | | 5´ | cgcgagctcGGATGCTCATAAACGGAAGG^1^ | | | cgcactagtATCTAAACGTCAAGATTTCGT | | |
|  |  | 3´ | cgcggtaccTGTCGCGTCATGAAAATGAT | | | ggcggtcgacTCGTACAATTATTTATTAATTTATTC | | |
| SpIL17-4a | | 5´ | cgcgagctcGATGTTTTGAAGGCATTTCT | | | cgcactagtGGCTGTGATCAAAAATAAGAAGGA | | |
|  | | 3´ | ggcggtcgacAAAACCAACGTTTCCTTATC | | | cgcggtaccTTTCTCCTCGATCACCATC | | |

^1^ Restriction digest sequences used for cloning are shown in lower case.
