# Supplementary material for: IL17 factors are early regulators in the gut epithelium during inflammatory response to Vibrio in the sea urchin larva
Source: eLife. 2017 Apr 27;6:e23481. doi: 10.7554/eLife.23481 (PMC5457136; doi:10.7554/eLife.23481)
Supplement: Supplementary file 2. — DOI: http://dx.doi.org/10.7554/eLife.23481.022 [file elife-23481-supp2.docx]

**Supplementary File 2: Genomic coordinates for the echinoderm *IL17* genes**

| ***S. purpuratus IL17 genes (v4.2)*** | | | | | | | |  |
| --- | --- | --- | --- | --- | --- | --- | --- | --- |
| *Group* | *Gene* | *Scaffold* | *Strand* | | *Exon positions* | *SPU_ID* | |  |
| 1 | 1a | 1147 | - | | 162335-162528, 160298-161609 |  | |  |
|  | 1b | 1147 | - | | 154949-155142, 153317-154629 |  | |  |
|  | 1c | 1147 | - | | 149635-149827, 147920-149298 |  | |  |
|  | 1d | 1147 | - | | 134122-134314, 132411-133791 |  | |  |
|  | 1e | 1147 | - | | 126987-127179, 125321-126650 | SPU_019350 | |  |
|  | 1f | 1147 | - | | 122889-123082, 121240-122552 |  | |  |
|  | 1g | 1147 | - | | 117526-117717, 115773-117186 | SPU_019351 | |  |
|  | 1h | 1147 | + | | 110116-110426, 111036-112820 | SPU_019349 | |  |
|  | 1i | 30714 | - | | 23011-23117, 22128-22691 |  | |  |
|  | 1j | 30714 | - | | 11426-11991 |  | |  |
|  | 1k | 30714 | - | | 6938-6943, 6047-6607 | SPU_012844 | |  |
| 2 | 2a | 1460 | + | | 23597-23885, 26211-26449, 27309-27515 | SPU_030200 | |  |
| 3 | 3a | 1460 | + | | 47414-47645, 51670-51917, 52712-52831 | SPU_022838 | |  |
| 4 | 4a | 1460 | - | | 105142-105204, 104534-104841, 100247-102081 |  | |  |
|  | 4a´ | 1460 | - | | 106329-106518, 104534-104841, 100247-102081 |  | |  |
| 5 | 5a | 2239 | + | | 61127-61717 | SPU_030204 | |  |
|  | 5b | 2239 | - | | 51362-51895 | SPU_030203 | |  |
| 6 | 6a | 1460 | + | | 59514-60087 | SPU_030184 | |  |
|  | 6b | 1460 | + | | 79593-80166 | SPU_030186 | |  |
|  | 6c | 1460 | + | | 85699-86269 | SPU_030185 | |  |
| 7 | 7a | 2076 | + | | 87460-87788, 89500-89655, 90306-90381 | SPU_030196 | |  |
| 8 | 8a | 2076 | + | | 76918-77288, 77654-77806, 78452-78536 | SPU_030197 | |  |
| 9 | 9a | 1219 | - | | 27549-28000, 26353-26508, 25489-25779 | SPU_027904 | |  |
|  | 9b | 1219 | - | | 14037-14488, 12844-12999, 11782-12072 | SPU_030188 | |  |
|  | 9c | 2076 | + | | 20714-21123, 22120-22275, 22824-23113 | SPU_030193 | |  |
|  | 9d | 2076 | - | | 45125-45564, 44403-44558, 43564-43810 | SPU_030191 | |  |
|  | 9e | 2076 | - | | 59309-60117, 58592-58747, 57780-58063 | SPU_030198 | |  |
|  | 9f | 74 | + | | 945549-946339, 947873-948028, 948599-948889 | SPU_030202 | |  |
|  | 9g | 950 | - | | 114716-114975, 113627-113782, 112757-113046 | SPU_030192 | |  |
| 10 | 10a | 1147 | + | | 170281-170802 | SPU_030199 | |  |
|  | 10b | 217 | + | | 21827-21841, 22360-22890 | SPU_005983 | |  |
| Pseudo-genes | p1 | 74 | + | | 1042446-1042736, 1043419-1043574, 1044254-1044543 |  | |  |
|  | p2 | 1460 | + | | 68005-68456 |  | |  |
|  | p3 | 2076 | + | | 9819-10109 |  | |  |
|  | p4 | 2076 | + | | 30415-30695, 35108-35270, 35813-36112 |  | |  |
|  | p5 | 30714 | - | | 17686-17809,16358-17355 |  | |  |
|  |  |  |  | |  |  | |  |
| ***Lytechinus variegatus IL17 genes (v2.2)*** | | | | | |  | |  |
| *Group* | *Gene* | *Scaffold* | *Strand* | | *Exon positions* | | |  |
| 1 | 1a | 1419 | + | | 58764-59243 | | |  |
|  | 1b | 1419 | - | | 64506-65054 | | |  |
| 2 | 2a | 13328 | - | | 24481-24805, 16682-16914, 14616-14678 | | |  |
| 3 | 3a | 2586 | + | | 5448-5673, 6562-6792 | | |  |
|  | 3b | 2586 | + | | 8342-8567, 9123-9400 | | |  |
| 4 | 4a | 2586 | - | | 54488-54825, 51033-51330 | | |  |
| 5 | 5a | 10 | + | | 187101-187652 | | |  |
|  | 5b | 10 | - | | 171157-171684 | | |  |
| 6 | 6a | 2586 | + | | 37830-38408 | | |  |
|  | 6b | 2586 | + | | 27138-38408 | | |  |
| 7 | 7a | 1097 | - | | 42201-42553, 37995-38150, 33581-33713 | | |  |
| 8 | 8a | 14109 | + | | 15636-16009, 16796-16949 | | |  |
| 9 | 9a | 1097 | + | | 113927-114135, 114991-115146, 116656-116728 | | |  |
|  | 9a´ | 1097 | + | | 112671-112879, 114991-115146, 116656-116728 | | |  |
| 10 | 10a | 1419 | + | | 50061-50549 | | |  |
|  |  |  |  | |  | |  |  |
| ***Eucidaris tribuloides IL17 genes (v1.0)*** | | | | | | | |  |
| *Group* | *Gene* | *Scaffold* | | *Strand* | *Exon positions* | | |  |
| 1 | 1a | JZLH01S0325643.1 | | - | 39523-40077 | | |  |
|  | 1b | JZLH01S0262536.1 | | + | 34356-34364, 35336-35893 | | |  |
|  | 1c | JZLH01S0077620.1 | | - | 12909-13466 | | |  |
|  | 1d | JZLH01S0599856.1 | | + | 36102-36659 | | |  |
|  | 1e | JZLH01S0231559.1 | | + | 8087-8641 | | |  |
|  | 1f | JZLH01S0620886.1 | | + | 52555-52632,48390-48944 | | |  |
| 2 | 2a | JZLH01S0079165.1 | | - | 39098-39203, 36842-37048, 34877-35153, 32035-32308 | | |  |
| 4 | 4a | JZLH01S0032189.1 | | - | 49845-49935, 49527-49761, 47905-47998, 45348-45490, 44604-44901 | | |  |
|  | 4b | JZLH01S0428811.1 | | - | 747-1388 | | |  |
|  | 4c | JZLH01S0334033.1 | | + | 9889-10527 | | |  |
| 5 | 5a | JZLH01S0084264.1 | | + | 92498-93040 | | |  |
|  | 5b | JZLH01S0084264.1 | | - | 108349-108894 | | |  |
| 6 | 6a | JZLH01S0032189.1 | | + | 35523-36158 | | |  |
|  | 6b | JZLH01S0500496.1 | | + | 8502-9029 | | |  |
|  | 6c | JZLH01S0007035.1 | | - | 14020-14547 | | |  |
| 7 | 7a | JZLH01S0122401.1 | | - | 8564-8895, 7324-7473, 6144-6210 | | |  |
|  | 7b | JZLH01S0074308.1 | | - | 12756-13087, 11546-11695, 10321-10387 | | |  |
| 8 | 8a | JZLH01S0618672.1 | | + | 22134-22642, 23168-23317, 24676-24751 | | |  |
|  | 8b | JZLH01S0017208.1 | | - | 8180-8604, 6992-7141, 6439-6514 | | |  |
| Novel | N1 | JZLH01S0411418.1 | | + | 1239-1479, 2864-2947 | | |  |
|  | N2 | JZLH01S0548846.1 | | + | 50279-50694, 53505-53654, 54352-54415 | | |  |
|  | N3 | JZLH01S0092465.1 | | - | 1282-1709, 469-631 | | |  |
|  | N4 | JZLH01S0233149.1 | | - | 66392-66632, 63887-64019, 62616-62742 | | |  |
|  |  |  |  | |  | | | |
| ***Patiria miniata IL17 genes (v1.0)*** | | | | | | |  | |
|  | *Gene* | *Scaffold* | *Strand* | | *Exon positions* | | | |
|  | 1 | 2072 | + | | 18011-18145, 20151-20612 | | | |
|  | 2 | 2072 | + | | 35047-35655 | | | |
|  | 3 | 2072 | + | | 41799-42338 | | | |
|  | 4 | 2072 | + | | 43728-44261 | | | |
|  | 5 | 2072 | - | | 47616-47772, 49793-50180 | | | |
|  | 6 | 2072 | + | | 66274-66792 | | | |
|  | 7 | 2072 | - | | 79254-79763 | | | |
|  | 8 | 569 | + | | 87721-88145, 88662-88764, 89299-89385 | | | |
|  | 9 | 569 | - | | 96064-96135, 97246-97355, 97928-98355 | | | |
|  | 10 | 569 | + | | 103247-103659, 104146-104248, 104751-104882 | | | |
|  | 11 | 235 | - | | 1709-1871, 2364-2506 | | | |
|  | 12 | 3619 | - | | 36391-36585, 37034-37296 | | | |
