# Supplementary material for: IL17 factors are early regulators in the gut epithelium during inflammatory response to Vibrio in the sea urchin larva
Source: eLife. 2017 Apr 27;6:e23481. doi: 10.7554/eLife.23481 (PMC5457136; doi:10.7554/eLife.23481)
Supplement: Supplementary file 3. — DOI: http://dx.doi.org/10.7554/eLife.23481.023 [file elife-23481-supp3.docx]

**Supplementary File 3: SEFIR domain-containing proteins**

| **Uniprot ID** | **Gene name** | **Species** |
| --- | --- | --- |
| **ACT1** |  |  |
| CIKS_HUMAN | TRAF3IP2 C6orf2 C6orf4 C6orf5 C6orf6 | *Homo sapiens* |
| Q8N7N6_MOUSE | Traf3ip2 mCG_1382 | *Mus musculus* |
| G3HMS0_CRIGR | I79_012042 | *Cricetulus griseus* |
| A0A091DHE8_FUKDA | H920_08094 | *Fukomys damarensis* |
| L5JPG6_PTEAL | PAL_GLEAN10018615 | *Pteropus alecto* |
| G1SQH7_RABIT | TRAF3IP2 | *Oryctolagus cuniculus* |
| L5M3H8_MYODS | MDA_GLEAN10019010 | *Myotis davidii* |
| V8PFR5_OPHHA | TRAF3IP2 L345_01315 | *Ophiophagus hannah* |
| Q28HY6_XENTR | traf3ip2 traf3ip3 TEgg012b24.1-001 | *Xenopus tropicalis* |
| C3Z1H5_BRAFL | BRAFLDRAFT_225945 | *Branchiostoma floridae* |
| C3Z1H6_BRAFL | BRAFLDRAFT_82312 | *Branchiostoma floridae* |
| K1QKG1_CRAGI | CGI_10027691 | *Crassostrea gigas* |
| K1QRW7_CRAGI | CGI_10027692 | *Crassostrea gigas* |
|  |  |  |
| **IL17RA** |  |  |
| I17RA_HUMAN | IL17RA IL17R | *Homo sapiens* |
| H2QL69_PANTR | IL17RA | *Pan troglodytes* |
| G1R8G7_NOMLE | IL17RA | *Nomascus leucogenys* |
| F6Z8G1_MACMU | IL17RA | *Macaca mulatta* |
| A0A096NVX3_PAPAN | IL17RA | *Papio anubis* |
| A0A0D9RPW9_CHLSB | IL17RA | *Chlorocebus sabaeus* |
| F7GCI2_CALJA | IL17RA | *Callithrix jacchus* |
| H0XIV2_OTOGA | IL17RA | *Otolemur garnettii* |
| I17RA_MOUSE | Il17ra Il17r | *Mus musculus* |
| D4A740_RAT | Il17ra | *Rattus norvegicus* |
| M3Z325_MUSPF | IL17RA | *Mustela putorius furo* |
| H0W275_CAVPO | IL17RA | *Cavia porcellus* |
| M3WVX7_FELCA | IL17RA | *Felis catus* |
| F1Q490_CANLF | IL17RA | *Canis lupus familiaris* |
| F1N2C5_BOVIN | IL17RA | *Bos taurus* |
| F6XT99_HORSE | IL17RA | *Equus caballus* |
| G1TZF2_RABIT | IL17RA | *Oryctolagus cuniculus* |
| G1LBG2_AILME | IL17RA | *Ailuropoda melanoleuca* |
| G1P0N9_MYOLU | IL17RA | *Myotis lucifugus* |
| G3SZ94_LOXAF | IL17RA | *Loxodonta africana* |
| F6XBR1_MONDO | IL17RA | *Monodelphis domestica* |
| F7BLH0_ORNAN | IL17RA | *Ornithorhynchus anatinus* |
| G3VBP9_SARHA | IL17RA | *Sarcophilus harrisii* |
| U3IYH9_ANAPL | IL17RA | *Anas platyrhynchos* |
| U3JK24_FICAL | IL17RA | *Ficedula albicollis* |
| G3UUX1_MELGA | IL17RA | *Meleagris gallopavo* |
| H0ZQK2_TAEGU | IL17RA | *Taeniopygia guttata* |
| G1KY25_ANOCA | IL17RA | *Anolis carolinensis* |
| V8P818_OPHHA | IL17RA L345_04043 | *Ophiophagus hannah* |
| H3B9K7_LATCH | IL17RA | *Latimeria chalumnae* |
| W5K1G6_ASTMX | IL17RA | *Astyanax mexicanus* |
| F2Z4X3_DANRE | il17ra1a | *Danio rerio* |
| W5NGW9_LEPOC | IL17RA | *Lepisosteus oculatus* |
| A0A087XLN9_POEFO | IL17RA | *Poecilia formosa* |
|  |  |  |
| **IL17RB** |  |  |
| I17RB_HUMAN | IL17RB CRL4 EVI27 IL17BR NQ2501/PRO19612 | *Homo sapiens* |
| G3QHE5_GORGO | IL17RB | *Gorilla gorilla gorilla* |
| G1R6J8_NOMLE | IL17RB | *Nomascus leucogenys* |
| F6SIU7_MACMU | IL17RB | *Macaca mulatta* |
| A0A096MY23_PAPAN | IL17RB | *Papio anubis* |
| A0A0D9RKH6_CHLSB | IL17RB | *Chlorocebus sabaeus* |
| F7E499_CALJA | IL17RB | *Callithrix jacchus* |
| H0XGG1_OTOGA | IL17RB | *Otolemur garnettii* |
| D3Z4T0_MOUSE | Il17rb | *Mus musculus* |
| B5DF15_RAT | Il17rb Il17rb_predicted rCG_42317 | *Rattus norvegicus* |
| I3LWR8_ICTTR | IL17RB | *Ictidomys tridecemlineatus* |
| M3YZG2_MUSPF | IL17RB | *Mustela putorius furo* |
| H0VS75_CAVPO | IL17RB | *Cavia porcellus* |
| M3W4E6_FELCA | IL17RB | *Felis catus* |
| J9P1Q7_CANLF | IL17RB | *Canis lupus familiaris* |
| A3KN55_BOVIN | IL17RB | *Bos taurus* |
| F6SPF8_HORSE | IL17RB | *Equus caballus* |
| G1T5J5_RABIT | IL17RB | *Oryctolagus cuniculus* |
| W5Q2V7_SHEEP | IL17RB | *Ovis aries* |
| F1SH81_PIG | IL17RB | *Sus scrofa* |
| G1L6L9_AILME | IL17RB | *Ailuropoda melanoleuca* |
| G3T6Y8_LOXAF | IL17RB | *Loxodonta africana* |
| F6Q0J4_MONDO | IL17RB | *Monodelphis domestica* |
| U3IVR2_ANAPL | IL17RB | *Anas platyrhynchos* |
| G1KLE5_ANOCA | IL17RB | *Anolis carolinensis* |
| V8NY88_OPHHA | IL17RB L345_07211 | *Ophiophagus hannah* |
| H3ARE8_LATCH | IL17RB | *Latimeria chalumnae* |
| W5N9Y1_LEPOC | IL17RB | *Lepisosteus oculatus* |
|  |  |  |
|  |  |  |
| **IL17RC** |  |  |
| H2QM12_PANTR | IL17RC | *Pan troglodytes* |
| G3RQ92_GORGO | IL17RC | *Gorilla gorilla gorilla* |
| H2PA42_PONAB | IL17RC | *Pongo abelii* |
| A0A096N591_PAPAN | IL17RC | *Papio anubis* |
| F7CBX8_CALJA | IL17RC | *Callithrix jacchus* |
| H0X4Z9_OTOGA | IL17RC | *Otolemur garnettii* |
| I17RC_MOUSE | Il17rc | *Mus musculus* |
| D3ZIM0_RAT | Il17rc | *Rattus norvegicus* |
| I3LZW7_ICTTR | IL17RC | *Ictidomys tridecemlineatus* |
| H0V8D0_CAVPO | IL17RC | *Cavia porcellus* |
| M3WKA1_FELCA | IL17RC | *Felis catus* |
| E2RGZ1_CANLF | IL17RC | *Canis lupus familiaris* |
| E1BPP5_BOVIN | IL17RC | *Bos taurus* |
| G1U3Z7_RABIT | IL17RC | *Oryctolagus cuniculus* |
| W5P675_SHEEP | IL17RC | *Ovis aries* |
| F1SQE0_PIG | IL17RC | *Sus scrofa* |
| G1MCL8_AILME | IL17RC | *Ailuropoda melanoleuca* |
| G1P6D4_MYOLU | IL17RC | *Myotis lucifugus* |
| G3UD56_LOXAF | IL17RC | *Loxodonta africana* |
| F7D217_MONDO | IL17RC | *Monodelphis domestica* |
| F7A8Y8_ORNAN | IL17RC | *Ornithorhynchus anatinus* |
| U3KEW0_FICAL | IL17RC | *Ficedula albicollis* |
| G1KI08_ANOCA | IL17RC | *Anolis carolinensis* |
| M4ASQ8_XIPMA | IL17RC | *Xiphophorus maculatus* |
| H2ZU29_LATCH | IL17RC | *Latimeria chalumnae* |
| A0A087Y9C5_POEFO | IL17RC | *Poecilia formosa* |
| H2TK03_TAKRU | IL17RC | *Takifugu rubripes* |
| Q4RJU4_TETNG | IL17RC GSTENG00033274001 | *Tetraodon nigroviridis* |
|  |  |  |
| **IL17RD** |  |  |
| I17RD_HUMAN | IL17RD IL17RLM SEF UNQ6115/PRO20026 | *Homo sapiens* |
| H2QMT9_PANTR | IL17RD | *Pan troglodytes* |
| G3QQP1_GORGO | IL17RD | *Gorilla gorilla gorilla* |
| H2PAG0_PONAB | IL17RD | *Pongo abelii* |
| G1R6M3_NOMLE | IL17RD | *Nomascus leucogenys* |
| F7HIX6_MACMU | IL17RD | *Macaca mulatta* |
| A0A0D9RK31_CHLSB | IL17RD | *Chlorocebus sabaeus* |
| F7EEM3_CALJA | IL17RD | *Callithrix jacchus* |
| H0WX16_OTOGA | IL17RD | *Otolemur garnettii* |
| I17RD_MOUSE | Il17rd Il17rlm Sef | *Mus musculus* |
| D3ZGQ0_RAT | Il17rd | *Rattus norvegicus* |
| I3LWH7_ICTTR | IL17RD | *Ictidomys tridecemlineatus* |
| M3YZI8_MUSPF | IL17RD | *Mustela putorius furo* |
| H0VEY7_CAVPO | IL17RD | *Cavia porcellus* |
| M3W0K4_FELCA | IL17RD | *Felis catus* |
| F1Q0W5_CANLF | IL17RD | *Canis lupus familiaris* |
| E1BP15_BOVIN | IL17RD | *Bos taurus* |
| F6W2W1_HORSE | IL17RD | *Equus caballus* |
| G1T6M7_RABIT | IL17RD | *Oryctolagus cuniculus* |
| W5PZ60_SHEEP | IL17RD | *Ovis aries* |
| K7GPZ0_PIG | IL17RD | *Sus scrofa* |
| D2GWZ7_AILME | IL17RD PANDA_001376 | *Ailuropoda melanoleuca* |
| G1NZF4_MYOLU | IL17RD | *Myotis lucifugus* |
| G3T9K3_LOXAF | IL17RD | *Loxodonta africana* |
| F7CYL0_ORNAN | IL17RD | *Ornithorhynchus anatinus* |
| U3I5B9_ANAPL | IL17RD | *Anas platyrhynchos* |
| U3K1F2_FICAL | IL17RD | *Ficedula albicollis* |
| I17RD_CHICK | IL17RD SEF | *Gallus gallus* |
| G1N3D6_MELGA | IL17RD | *Meleagris gallopavo* |
| H0Z9Y6_TAEGU | IL17RD | *Taeniopygia guttata* |
| G1KEK5_ANOCA | IL17RD | *Anolis carolinensis* |
| V8PET4_OPHHA | IL17RD L345_01760 | *Ophiophagus hannah* |
| F6ZRR0_XENTR | il17rd | *Xenopus tropicalis* |
| H3ANI2_LATCH | IL17RD | *Latimeria chalumnae* |
| I17RD_DANRE | il17rd cb208 sef | *Danio rerio* |
| C3YAT4_BRAFL | BRAFLDRAFT_102559 | *Branchiostoma floridae* |
| K1QKP5_CRAGI | CGI_10002512 | *Crassostrea gigas* |
|  |  |  |
| **IL17RE** |  |  |
| F7HK29_MACMU | IL17RE | *Macaca mulatta* |
| F6W560_CALJA | IL17RE | *Callithrix jacchus* |
| I17RE_MOUSE | Il17re | *Mus musculus* |
| I17RE_RAT | Il17re | *Rattus norvegicus* |
| M3Z0C4_MUSPF | IL17RE | *Mustela putorius furo* |
| E2RGV8_CANLF | IL17RE | *Canis lupus familiaris* |
| I3L6L2_PIG | IL17RE | *Sus scrofa* |
| G1P064_MYOLU | IL17RE | *Myotis lucifugus* |
| F7GGQ5_MONDO | IL17RE | *Monodelphis domestica* |
| G1KI13_ANOCA | IL17RE | *Anolis carolinensis* |
| F7CXC1_XENTR | il17re | *Xenopus tropicalis* |
|  |  |  |
|  |  |  |
| **Other** |  |  |
| C3YAT5_BRAFL | BRAFLDRAFT_102558 | *Branchiostoma floridae* |
| C3Z2Q8_BRAFL | BRAFLDRAFT_66438 | *Branchiostoma floridae* |
| K1PLG7_CRAGI | CGI_10021486 | *Crassostrea gigas* |
